# Failure to follow up abnormal test results associated with cervical cancer in primary and ambulatory care: a systematic review

**DOI:** 10.1186/s12885-023-11082-z

**Published:** 2023-07-12

**Authors:** Javiera Martinez-Gutierrez, Sophie Chima, Lucy Boyd, Asma Sherwani, Allison Drosdowsky, Napin Karnchanachari, Vivien Luong, Jeanette C. Reece, Jon Emery

**Affiliations:** 1 Centre for Cancer Research and Department of General Practice, University of Melbourne, Melbourne, Australia; 2 Department of Family Medicine. Pontificia, Universidad Católica de Chile, Santiago, Chile; 3 Melbourne School of Population and Global Health, Centre for Health Policy, The University of Melbourne, Melbourne, Australia; 4 Neuroepidemiology Unit, Centre for Epidemiology and Biostatistics, Melbourne School of Population and Global Health, The University of Melbourne, Melbourne, Australia

**Keywords:** Inadequate follow-up, Abnormal test results, Primary care, Cervical cancer, Human papilloma virus

## Abstract

**Background:**

Cervical cancer is a preventable and treatable form of cancer yet continues to be the fourth most common cancer among women globally. Primary care is the first point of contact most patients have with health services and is where most cancer prevention and early detection occur. Inadequate follow-up of abnormal test results for cervical abnormalities in primary care can lead to suboptimal patient outcomes including higher mortality and decreased quality of life.

**Aims:**

To explore the magnitude of and factors associated with, inadequate follow-up of test results for cervical abnormalities in primary and ambulatory care.

**Methods:**

MEDLINE, Embase, Cochrane Library and CINAHL were searched for peer-reviewed literature from 2000–2022, excluding case-studies, grey literature, and systematic reviews. Studies were included if they reported on patients aged ≥ 18 years with no previous cancer diagnosis, in a primary care/ambulatory setting. Risk of bias was assessed using the Joanna Briggs Institute Critical appraisal checklists, appropriate to the study design. A segregated methodology was used to perform a narrative synthesis, maintaining the distinction between quantitative and qualitative research.

**Results:**

We included 27 publications reporting on 26 studies in our review; all were conducted in high-income countries. They included 265,041 participants from a variety of ambulatory settings such as family medicine, primary care, women’s services, and colposcopy clinics. Rates of inadequate follow-up ranged from 4 to 75%. Studies reported 41 different factors associated with inadequate follow-up. Personal factors associated with inadequate follow-up included younger age, lower education, and socioeconomic status. Psychological factors were reported by only 3/26 studies and 2/3 found no significant association. System protective factors included the presence of a regular primary care provider and direct notification of abnormal test results.

**Discussion:**

This review describes inadequate follow-up of abnormal cervical abnormalities in primary care. Prevalence varied and the evidence about causal factors is unclear. Most interventions evaluated were effective in decreasing inadequate follow-up. Examples of effective interventions were appointment reminders via telephone, direct notification of laboratory results, and HPV self-sampling. Even though rates of cervical cancer have decreased over the years, there is a lack of information on factors affecting follow-up in primary care and ambulatory settings, particularly in low and middle-income countries. This information is crucial if we are to achieve WHO’s interim targets by 2030, and hope to avert 62 million cervical cancer deaths by 2120.

**Trial registration:**

PROSPERO ID CRD42021250136.

**Supplementary Information:**

The online version contains supplementary material available at 10.1186/s12885-023-11082-z.

## Introduction

### Cervical cancer globally

Cervical cancer is the fourth most common cancer among women globally, with an estimated 604,100 new cases and 341,831 deaths in 2020 [1]. Even though preventable and treatable, cervical cancer continues to affect women and people with a cervix, is associated with substantial impacts on quality of life, and causes extreme suffering in late-stage disease. In 2020, the World Health Organisation (WHO) released a global strategy with a vision to eliminate cervical cancer as a public health problem [2]. The strategy proposes interim targets focusing on primary prevention (vaccination against HPV), secondary prevention (screening and treatment of pre-cancerous lesions), tertiary prevention (diagnosis and treatment of pre-cancer and invasive cervical cancer), and palliative care. Modelling has shown hundreds of thousands of lives would be saved if interim targets are met worldwide by 2030 and millions saved by 2120 [3].

### Biology of cervical cancer and progression

The primary etiologic factor for over 95% of cervical cancers is persistent infection with high-risk Human Papilloma Virus (hrHPV) [4]. There are many strains of HPV, with types 16 and 18 accounting for approximately 70% of cervical cancer cases, and an additional 10–15 strains responsible for the rest [5]. Not all infections will lead to cervical lesions, but persistent HPV infection can lead to changes in the cervical cells, and these can develop into cervical cancer over decades, hence the importance of timely follow-up [5]. High-performance cervical screening tests can detect changes to cells as well as the presence and type of HPV infection prior to any cell changes. Results of cervical screening tests can be ‘normal’ (‘negative’): no HPV or cervical cell changes detected, or abnormal (‘positive’): HPV positive (presence of HPV infection) or abnormal cells (LSIL/HSIL/ASCUS—low/high grade squamous intraepithelial lesion or atypical squamous cells of undetermined significance). Results that are considered ‘abnormal’ or ‘positive’ require follow-up and/or treatment to avoid progression to invasive cervical cancer. Examples of follow-up can be via second-round testing, a colposcopy with/without a biopsy and, if necessary, excision treatment, [6] yet specific guidelines for follow-up will vary depending on the country and health care system.

### Cervical cancer and primary care

Delays in the follow-up of pre-cancerous lesions in primary care, can lead to delays in cancer diagnosis, which, in turn, may result in suboptimal cancer outcomes such as higher mortality and decreased quality of life [7]. In many developed countries, primary care accounts for more than 80% of all health consultations in the general population and acts as gatekeeper for many interventions and specialist consultations [8, 9]. Defined by the WHO as the “best way to provide health care services to everyone, everywhere,” primary care is described as the most efficient way to achieve health for all [10]. Early detection, appropriate monitoring and treatment of positive or abnormal cervical screening results can lead to improved outcomes, and a reduction in adverse effects [11]. Importantly, adherence to cervical cancer guidelines can directly impact patient survival [12].

Adequate follow-up may be difficult to define, as there are multiple pathways. The WHO, in their “Guidelines For Screening and Treatment of Cervical Pre-cancer Lesions for Cervical Cancer Prevention” recommends two main approaches after a positive screening test: The “screen-and-treat approach”, where a decision is made to treat immediately after one positive test result; or the “screen, triage and treat approach”, where a second test may be required to decide treatment [6]. Further follow-up can be partial genotyping, colposcopy, visual inspection with acetic acid (VIA) or cytology [6].

There are different ways to define inadequate follow-up”; it may be due to a delay in performing a second test or treatment, or it may be defined as follow-up using an inappropriate screening test according to relevant guidelines (i.e., Pap smear instead of colposcopy). A range of factors leading to inadequate follow-up have been described. For example, a systematic review of studies published between 1985 and 1999 reported patient factors such as lack of social support, lack of understanding, and fear [13]. Health care system factors include inconvenient clinic hours, male providers, and insensitive staff. More recent systematic reviews reporting on adherence also mention social support, factors such as race and low socioeconomic status (SES) as well as provider/system factors [14–17]. However, current evidence from systematic reviews relies on data from earlier than 2010 and no reviews report exclusively on prevalence and factors associated with inadequate follow-up in primary and ambulatory care. Furthermore, primary care differs in each country’s local context, so it is crucial to understand the nuances of inadequate follow-up of abnormal cervical screening results in the ambulatory setting.

### Aim and justification for current review

The WHO global strategy provides a comprehensive approach to cervical cancer prevention and treatment [2]. However, the interim targets fail to address inadequate or failure to follow-up of abnormal test results, which may lead to suboptimal outcomes and unnecessary harm. This review aims to address two key questions in relation to inadequate follow-up of abnormal test results associated with cervical screening in primary care: i) what is the magnitude of the problem in primary and ambulatory care and; ii) what are the precursors or predictors associated with inadequate follow-up.

## Method

### Study design

The Preferred Reporting Items for Systematic Reviews and Meta-Analyses (PRISMA) criteria were used to conduct a narrative, systematic review [18]. The review was registered on PROSPERO (registration ID: CRD42021250136). Covidence systematic review software was used for title and abstract screening, full text review and data extraction. A meta-analysis was not performed, due to the heterogeneity of included studies.

### Search strategy

The search strategy (see appendix 1) was developed to include MeSH headings and word variations for the terms relating to ‘primary care, ‘follow-up’ and any screening and diagnostic tests relating to cervical cancer (e.g., Pap test, HPV, colposcopy). Searches were conducted across MEDLINE, EMBASE, the Cochrane Central Register of Controlled Trials (CENTRAL) and the Cumulative Index to Nursing and Allied Health Literature (CINAHL). Studies were included if they were published between 1 Jan 2000 and 17 March 2022. This timeline was selected as existing systematic reviews on related topics included studies from previous years, [13, 17] and we believed it relevant to assess follow-up behaviours over the last 20 years. Supplementary searching included a manual review of reference lists of included studies and citation tracking.

### Inclusion and exclusion criteria

According to the PICO framework, studies were included if they referred to female adult populations (≥ 18 years) in primary/ambulatory care (P), who had undergone any screening and diagnostic testing related to cervical cancer and had received an abnormal/positive test result (I) and had not been followed up adequately according to local or national guidelines (O). As all types of studies were included, some studies did not have a comparator and outcomes varied according to study type. We included studies involving females < 18 years only if outcome data could be extracted for patients > 18 years old separately. Studies reporting follow-up of abnormal results that may lead to cervical cancer, including those reporting tests for screening, diagnosis of other gynecological cancers were included. We excluded case studies, unpublished work and articles not in English. Studies reporting only on children and adolescents (< 18 years); people with an existing cancer diagnosis; and follow-up conducted in tertiary care were excluded. Studies which solely reported appropriate or timely follow-up and not explicitly inadequate follow-up were also excluded. This decision was made to avoid assuming that women that did not have adequate follow-up corresponded to inadequate follow-up, as these concepts are not necessarily the same since these women may have accessed follow-up care elsewhere. Screening and full-text review was performed by two independent authors and conflicts were reached by consensus. Where consensus was not reached, opinion of a third independent author was sought.

### Data collection

After studies were assessed against the inclusion and exclusion criteria, data were extracted from included studies, based on a standardized extraction template. Extracted data included study and participant characteristics; the number or percentage of women with abnormal test results that had inadequate follow-up; and barriers and facilitators to inadequate follow-up.

### Statistical analysis

We used SPSS version 29.0.0.0 (241) to conduct a descriptive analysis of prevalence and study characteristics. The heterogeneity of study designs and outcomes precluded any form of meta-analysis.

### Assessment of bias

The risk of bias of included studies were assessed using the Joanna Briggs Institute (JBI) Critical appraisal checklists, appropriate to the study design of the included studies [19]. Eight checklists were used, including the checklists for randomised controlled trials, quasi-experimental, cohort, cross-sectional, case–control, case series, qualitative and diagnostic accuracy studies. Studies that scored ≥ 80% were considered low risk of bias, between 60–80% a medium risk of bias, and < 60% were considered high risk [20]. Studies were not excluded based on their quality assessment scores [19].

Extraction and risk assessment were also performed by two independent authors and conflicts were reached by consensus. Where consensus was not reached, opinion of a third independent author was sought.

### Definition of inadequate follow up of cervical cancer screening in primary care

**Fig. 1:**
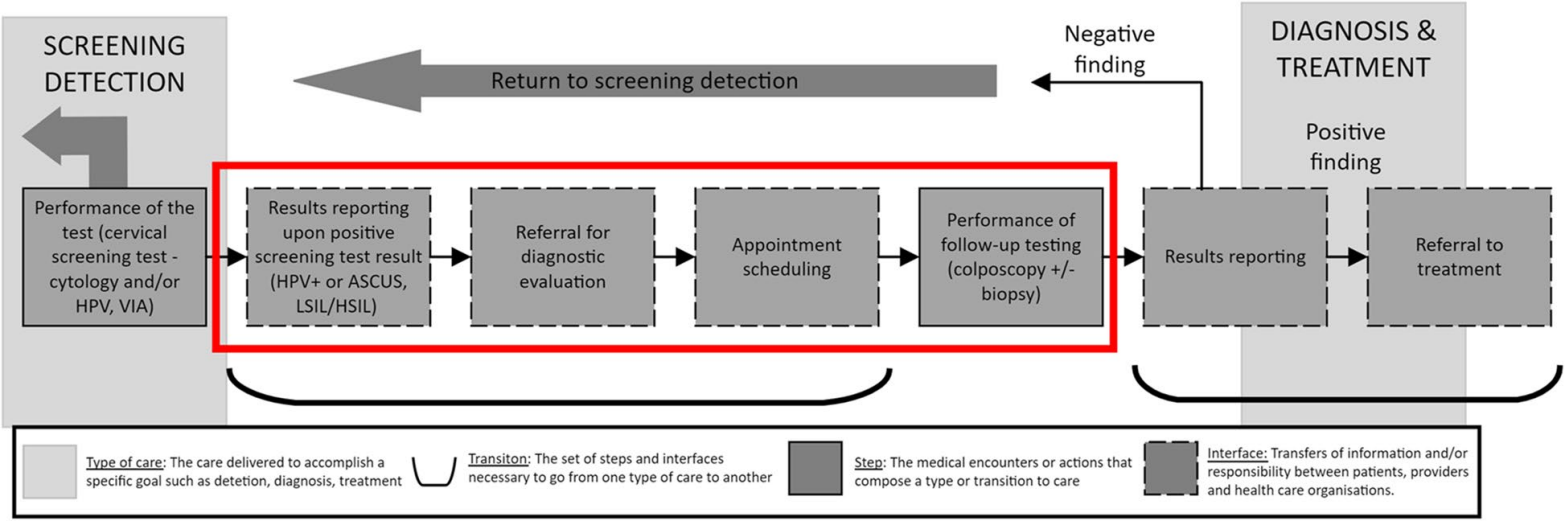
Steps and interfaces from screening to cancer diagnosis. Modified from Zapka et al., [16]

To identify studies that related to inadequate follow up of an abnormal test result for cervical cancer, we adapted Zapka et al. “Steps and Interfaces from screening to diagnosis” (Fig. 1) which defines this period as the moment after receiving a positive/ abnormal test result (HPV positive (presence of HPV infection) or abnormal cells (LSIL/HSIL/ASCUS—low/high grade squamous intraepithelial lesion or atypical squamous cells of undetermined significance) to just before a cancer diagnosis [16]. This period is comprised of several steps, including referral for a diagnostic evaluation, appointment scheduling, and a follow-up test (Pap, HPV, colposcopy etc.). We selected all studies that reported results related to this period.

## Results

### Overview

**Fig. 2:**
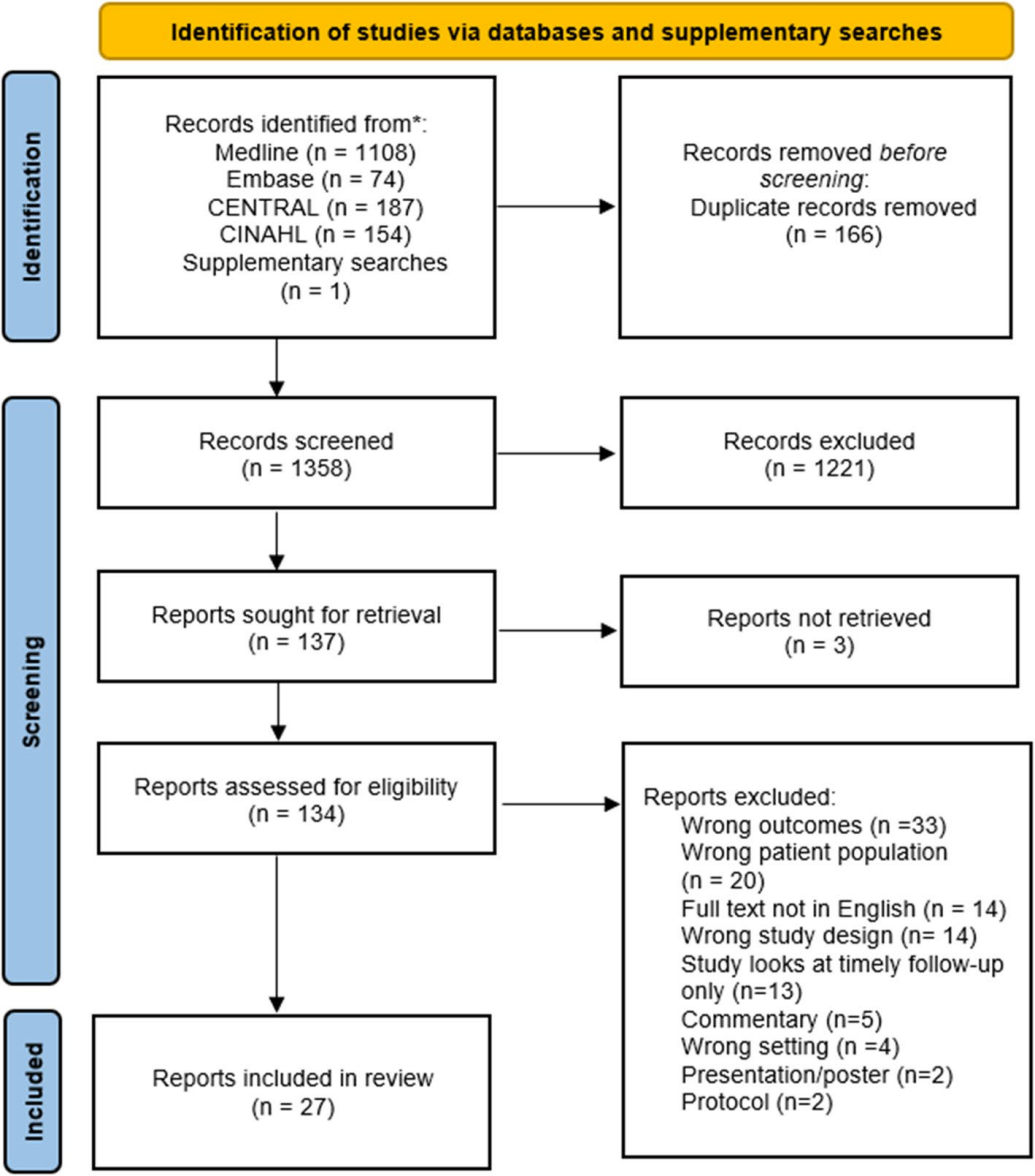
PRISMA flow diagram

The combined search strategies identified 1524 titles, of which 1358 title and abstracts were screened, and 134 full text articles reviewed for eligibility (Fig. 2). Twenty-seven reports, including 26 studies, met the inclusion criteria.

### Study characteristics

Characteristics of the included studies are described in Table 1. Twenty-seven publications were identified for inclusion in the review, including 26 primary studies [21–46] and one secondary analysis of the original study data looking at cervical cancer prevention behaviours of the studied population [47]. The results of the secondary analysis are reported together with the primary study as most study characteristics were the same. Using the JBI risk of bias checklists, we assessed 12 studies as low risk of bias, [22–24, 30, 33, 34, 38, 40, 41, 43, 44, 46] 10 with a moderate risk [21, 25, 26, 28, 29, 32, 35, 37, 42, 45] and four studies with a high risk of bias [27, 31, 36, 39]. The detailed quality assessment can be found in Appendix 2.

**Table 1:** Characteristics of included studies

Reference	Location	Study type	Study period	Tests studied	Setting	Number of sites	Inclusion criteria	Number of participants with abnormal test results	Age (years)	Risk of bias
Benard (2005) [21]	USA	Cohort	Jul 1991—Sept 2000	Pap smear and colposcopy	All screening services conducted within local health departments, community health centres, the practices of private physicians, and hospitals (as part of the NBCCEDP)	Unclear	Women from the national screening programme (NBCCEDP) who had a result of ASC-US or LSIL followed by a second ASC-US or LSIL	10,004	Range= 21–64	Medium
Breitkopf (2014) [22]	USA	RCT	June 2006—Nov 2010	Pap smear and colposcopy	Regional and Maternal Child Health Program clinics	6	Participants were Hispanic and non-Hispanic (black and white) women who attended one of the study clinics in southeast Texas for an appointment that included a Pap test	299	Range= 18–55	Low
Brewer (2021) [23]	New Zealand	RCT	June 2018 – May 2020	Pap smear, HPV, colposcopy	Family Medicine/Primary Care/General Practice	23	Never- and under-screened (no screening recorded for at least the last five years) Māori, ¯ Pacific, and Asian women in the Auckland area (Waitemata¯ and Auckland District Health Boards (DHBs))	24	Media*n* = 44.0 Range= 30–69	Low
Chase (2012) [24]	USA	Cohort	Nov 2006 -Dec 2007	Pap smear, colposcopy and HPV	University affiliated colposcopy clinic	1	All patients referred to the colposcopy clinic in the University of California (UC), Irvine, Family Health Center in Santa Ana for abnormal results for cervical cytology and/or high-risk HPV DNA infections	1046	Mean= 31 SD = 10.7	Low
Dunn (2013) [25]	Canada	Non-randomised experimental	Jan 2007—Sept 2010	Colposcopy	Ambulatory/specialist clinics	1	Women attending the sexual health clinic who were referred for colposcopy after an abnormal cytology test	685	Mean= 26	Medium
Engelstad (2005) [26]	USA	RCT	Sept 1999—Aug 2001	Pap smear	Hospital affiliated community clinic	1	Women were identified following receipt of acytological Pap smear result showing an abnormality of ASCUS or greater (clinic based)	348	Range = 18–74	Medium
Felix (2009) [27]	USA	Cross sectional	May 2006—Sept 2007	Pap smear	Clinics providing family planning services	Unclear	Clients of survey-responding family planning providers self-reporting an abnormal Pap smear	234	Range = 21–64	High
Fish (2013) [28]	USA	Case–control	Jan 2011—Jan 2012	Pap smear and colposcopy	Ambulatory/specialist clinics and hospital affiliated community clinics (within the Duke University Health System)	10	Women who had cervical cytopathology (Pap) testing at participating clinics that had a diagnosis of dysplasia or low-grade squamous intraepithelial lesion and were scheduled for a follow-up visit at 1 of 4 colposcopy clinics	184	Mean= 29.8 Range = 21–64	Medium
Gok (2010) [29]	The Netherlands	RCT	Dec 2006—Dec 2007	HPV, pap smear and colposcopy	Family Medicine/Primary Care/General Practice laboratories, and hospital	Unclear	Non-attendees to the Dutch screening programme living in the counties of Noord-Holland or Flevoland (*n* = 28073) who had received their screening invitation in 2005 were selected from the regional health council registry	757	Range = 30–60	Medium
Goldsmith (2008) [30]	UK	Qualitative	May 2005—April 2006	Pap smear, colposcopy and HPV	Screening centres and colposcopy clinic	4	Women that had attended for screening within 5–16 weeks upon study invitation and received either normal, inadequate, borderline or mildly dyskaryotic results. In one centre,women undergoing more frequent screening following a previous abnormal result (early recall group) were also included. In addition, two consecutive samples of women (50 in total) attending for colposcopy for the first time following screening were approached about participation by clinic staff	25	Range = 20–64	Low
Gultekin (2018) [31]	Türkiye	Cohort	Aug 2013—Oct 2014	HPV, pap smear and colposcopy	Family Medicine/Primary Care/General Practice and laboratories	Unclear	All adherent women invited to HPV based national screening programme	37,515	Mean= 45.6 Range = 30–65	High
Hui (2014) [32]	USA	Cross sectional	May 2006—June 2010	Colposcopy	Colposcopy clinic	1	Patients with an initial abnormal cervical cytology test result from the Temple University School of Medicine Women's Care Centre Colposcopy Clinic	210	Mean= 30 SD = 10.67	Medium
Hunt (2002) [33]	USA	Qualitative	Unclear	Pap smear	Cancer screening clinic	1	Mexican or Mexican American women who were older than age 40 years and had low incomes, who were classified as “lapsed” or “lost to follow-up” in the cancer screening clinic records	11	Range = 40–73	Low
Kristiansen (2017) [34]	Denmark	Cohort	Jan 2009—Aug 2013	Pap smear	Family Medicine/Primary Care/General Practice	2176	All cervical cytology samples with a recommended follow-up date 7 or 9 months earlier than 30 May 2014	124,244	Range = 23–64	Low
Kristiansen (2019) [35]	Denmark	RCT	Jan 2013 – June 2014	Pap smear and HPV	Family Medicine/Primary Care/General Practice	333	Women with a cervical cytology performed in a general practice during the study period that were recommended follow-up	11,833	Range = 23–64	Medium
Kupets (2014) [36]^**a**^	Canada	Cross sectional	Jan 2008—Dec 2009	Pap smear and colposcopy	Family Medicine/Primary Care/General Practice	Unclear	All abnormal cytology reports in CytoBase (contains cervical cytology samples collected in the community)	69,075	Range = 14 +	High
Lindau (2006) [37]	USA	Cohort	Jan—Dec 1999	Pap smear then colposcopy	Ambulatory primary care and HIV ob/gyn continuity of care clinics at a Chicago academic medical center	1	All women who presented to the ambulatory primary care and HIV ob/gyn continuity of care clinics at a Chicago academic medical centre	68	Range = 18–49	Medium
Loopik (2020) [38]	The Netherlands	Diagnostic accuracy	Dec 2018—Aug 2019	Pap smear, HPV and colposcopy	Family Medicine/Primary Care/General Practice and hospital affiliated clinics	2	High-risk HPV-positive women who attended the cervical cancer screening programme by self-sampling	1014	Range = 30–60	Low
Oladipo (2007) [39]	UK	RCT	July—Oct 2003	Colposcopy	Ambulatory/specialist clinic	1	All patients referred for colposcopic examination and treatment after abnormal cervical smear	189	Range = 20–65	High
Percac-Lima (2013) [40]	USA	Non-randomised experimental	Jan 2004 – April 2011	Pap smear and colposcopy	Colposcopy clinic and community health centre	2	Self-identified Latinas, with an abnormal Pap smear requiring colposcopy evaluation	786	Mean= 35 Range = 22–86	Low
Peterson (2003) [41]	USA	Cohort	Feb 1999—April 2000	Pap smear and colposcopy	Academic medical centre and neighbourhood health centres	8	All women 18 years or older, who had abnormal Pap smears in an academic medical centres’ computerized pathology database	423	Mean = 33 Range = 18 +	Low
Salyer (2021) [42]^b^ Salyer (2022) [47]^**b**^	USA	Cross-sectional	Mar 2019 – Jun 2020	Pap smear, HPV, colposcopy and biopsy	Family Medicine/Primary Care/General Practice, hospital affiliated clinic or community health centre	Unclear	Criminal legal-involved women from three US cities. All participants were women aged 18 or older and recruited from cohorts of women in ongoing, separate studies at each location. The three cohorts were merged to provide the investigative team with access to existing groups of hard-to reach women and follow a robust, multi-site cohort	58	Mean= 42.4 SD = 11.7 Range = 18–65	Medium
TOMBOLA Group (2009) [43]	Canada	RCT	Oct 1999—Oct 2002	Pap smear, colposcopy, HPV and biopsy	Family Medicine/Primary Care/General Practice and colposcopy clinics	3	Women living in Grampian, Tayside, and Nottingham, whose index cytology indicated mild dyskaryosis or borderline nuclear abnormality	4439	Range = 20–59	Low
Tse (2016) [44]	Hong Kong	Cohort	Jan 2005 -Dec 2006	Pap smear, colposcopy and HPV	Hospital and primary care	1	Patients attending the hospital colposcopy clinic and patients referred to the primary care centre for cervical smear screening within 3 months of colposcopy	833	Mean= 40.8 SD = 10.6	Low
UshaKiran (2002) [45]	UK	Case–control	1996–1998	Colposcopy	Ambulatory/specialist clinic	1	Women who were lost completely to follow-up were included in the study sample. A similar number of clinic attenders were randomly selected and used as a control group	685	Range = 20–52	Medium
Valdini (2001) [46]	USA	Case series	Jan 1997 – Dec 1998	Pap smear, colposcopy, HPV and LEEP/cone biopsies	Urban family/community health center	1	Inclusion for main series not outlined	52	Mean= 35 Range = 18–64	Low

^**a**^Outcome data extracted for patients > 20 years old only

^**b**^Study described in two publications

The included studies comprised seven RCTs [22, 23, 26, 29, 35, 39, 43] and seven cohort studies [21, 24, 31, 34, 37, 41, 44]. The remaining were cross-sectional [4], [27, 32, 36, 42] non-randomised experimental [2], [25, 40] case–control [2], [28, 45] qualitative [2], [30, 33] case series [1] [46] and diagnostic accuracy studies[1] [38]. Half of the studies were conducted in the USA [21, 22, 24, 26–28, 32, 33, 36, 37, 40–42, 46]. The remaining were conducted in Canada [3], [25, 36, 43] the UK [3], [30, 39, 45] Denmark [2], [34, 35] the Netherlands [2], [29, 38] New Zealand [1], [23] Hong Kong [1] [44] and Türkiye. [1] [31] The number of health care sites ranged from 1 to 2176, and the number of participants that had an abnormal test result requiring follow up ranged from 11 to 124,244. Even though the age of women included in the studies ranged from 14 to 86, for the purpose of this review, we only included data from women 18 years and older.

There were 7 population-based studies using data from large screening and testing datasets) [21, 29, 31, 34–36, 38]. The remining 19 were clinic-based. 7 studies included exclusively women from underserved populations (Latinas, low income, Maori and Criminal-legal involved) [22, 23, 26, 32, 33, 40, 42, 47] and 4 studies included only non-adherent women [23, 29, 33, 45].

While we grouped setting into four categories, due to the differences in health systems, we presented the setting from USA studies in more detail in Table 1. For non-USA studies, the setting was categorised into ambulatory/specialist clinics; primary care/general practice/family practice; hospital affiliated community clinic; and other/combination. Four studies were conducted exclusively in primary care/general practice/family practice [23, 34–36]. Three studies were conducted exclusively in ambulatory/specialist clinics [25, 39, 45]. The remaining studies were conducted in a combination of settings.

### Definition of inadequate follow-up

Inadequate follow-up was defined as either no evidence of further action (follow-up tests and/or appointments) or an inadequate action according to guidelines or local protocol. A detailed definition of inadequate follow-up for each study is presented in Table 2. The majority (87.5%) of the included studies had a clear definition for inadequate follow up of an abnormal test result [21–29, 33–45, 47]. Of these, 70% defined it as a failure to attend the following appointment either with their clinician or for a follow up test [22–25, 27–29, 33, 37, 39, 40, 42–45] and the remaining 30% defined it as no evidence of further testing in the electronic medical record (EMR) [21, 26, 34–36, 38, 41].

**Table 2:** Definitions and rates of inadequate follow up

Reference	Definition of inadequate follow-up	According to guidelines	Guidelines used for follow-up	Timeframe for follow up	Source used as evidence of follow-up	Study patient follow-up method	Rates of inadequate follow-up
Benard (2005) [21]	Inadequate follow up: Women with a 3^rd^ Pap after two abnormal Pap tests No follow up: No test after two abnormal Pap tests	Y	National Cancer Institute (NCI) interim guidelines (1994)	2 years after second abnormal Pap test	National Breast and Cervical Cancer Early Detection Program (NBCCEDP) database	N/A	Inadequate follow up: 28.3% No follow up: 27.7%
Breitkopf (2014) [22]	Failure to attend follow-up appointment Delay to attend Follow-up appointment (over 90 days)	Y	The optimum organization for the delivery of colposcopy service in Ontario: A systematic review. Journal of Lower Genital Tract Disease. (2010)	90 days from scheduled appointment and 18 months following abnormal test result for completeness of care	Prospective electronic medical record (EMR) review	Phone	Failure to attend: Active control: 25% Usual Care: 24% Standard care: 22% (*p* = 0.93) Delay in care (days) total: Intervention 58 +-75 Active Control: 69 + -72 Standard care: 54 + -75 (*p* = 0.75)
Brewer (2021) [23]	Failure to attend follow-up of HPV	Y	National Screening Guidelines (NSG) for cervical screening in New Zealand (2008)	Unclear for colposcopy (3 months for HPV screening)	Results mailed to study directly and other information obtained from National Cervical Screening Program Register and EMR	Mixed (In person or phone)	8% (2/24)
Chase (2012) [24]	No follow-up visit	Y	American Society of Colposcopy and Cervical Pathology (ASCCP) guidelines (2007)	2.5 to 14 months from initial appointment	Retrospective chart and/or EMR review	Mixed (phone and mail)	50% no-show rate
Dunn (2013) [25]	No consultation note or chart notation indicating a colposcopy visit	N	(-)	Six months and one day from referral date	Retrospective chart review	Phone	13% pre group 4% post group (p < 0.001)
Engelstad (2005) [26]	No repeat Pap smear and/or a colposcopy examination was performed	Y	Alameda County Medical Centre’s (ACMC’s) Highland Hospital (HGH) Guidelines	6 months period after her initial abnormal Pap smear	A centralized computerized tracking follow-up system (with information from the clinics computerized appointment system, clinics reports, and patients’ medical records	Mixed (In person or phone)	18% intervention (hosp) 20% control (primary care)
Felix (2009) [27]	Client self-report of not receiving care from a family planning (FP) provider or receiving elsewhere	N/A	(-)	N/A	Study Survey	Self-report	According to a patient survey 63.2% reported no treatment by a FP provider. Of those, 49.3% were not referred by a FP provider for follow-up
Fish (2013) [28]	Non-attendance to their scheduled appointment	N	(-)	N	Electronic clinic appointment logs	Mixed (Phone and mail)	N/A (population was non-adherent)
Gok (2010) [29]	A “non-attendee” was defined as a woman who neither responded to the regular invitation nor to a standard reminder	Y	Netherlands National Screening Program (2007)	6 months from invitation or reminder to attend follow up	Receipt of informed consent plus self-samples and Nationwide pathology database (PALGA)	Mail	Intervention group: 5.5% to 61.5% (depending on stage to follow up (from repeat HPV and Pap to colposcopy)
Goldsmith (2008) [30]	N/A	N/A	(-)	N/A	Women with a colposcopy appointment: List identified by clinic and waiting room recruitment	Mixed (Invitation package through clinic and phone)	N/A: Qualitative study on experiences with cervical screening communication
Gultekin (2018) [31]	Unclear	Y	Türkiye National Screening Program	Unclear	Regional clinics medical records	Mixed (Phone, mail and in person)	Not Reported
Hui (2014) [32]	Unclear	N/A	(-)	N/A	N/A	Mail	N/A Cross sectional studies about barriers to follow-up before their colposcopy appointment
Hunt (2002) [33]	Failure to attend a colposcopy appointment after 3 reminders, the last and 4th by certified letter	Y	Breast and Cervical Cancer Control Program Guidelines based on American Cancer Society Guidelines (1992)	N	Clinics’ records	Mixed (Phone, mail and in person)	N/A Qualitative study on factors affecting incomplete follow-up
Kristiansen (2017) [34]	No new cervical cytology or histology was registered after recommended follow-up	Y	Danish National Screening Programme and follow-up recommendations	6 months after reminder follow- up depending on period of recommendation (Within 3 m, at 3, 6, 9 and 12 months from initial cytology)	Danish Pathology Data Bank (DPDB)	N/A	6 months (before) 64% (After) 62% (OR: 0.94 (0.90 to 0.97)) 3 months: (before) 62% (after) 60% (OR: 0.94 (0.90 to 0.97)) Within 3 months, (before) 16% (after) 16% (OR: 0.99 (0.95 to 1.02))
Kristiansen (2019) [35]	Absence of a new cervical cytological or histological sample (i.e. including biopsies) at different relevant time points	Y	Danish National Screening Programme and follow-up recommendations	3, 6 or 12 months after initial cytology	Danish Pathology Data Bank (DPDB)	Mail	No timely follow up 47.2% (control) No timely follow up 42.9% (intervention) (*p* < 0.005) No follow up one month after recommended 35.0% (control) No follow up one month after recommended (intervention) 31.4% (*p* < 0.005)
Kupets (2014) [36] ^a^	Direct colposcopy without repeat cytology (inadequate) OR no evidence of colposcopy after cytology (inadequate). Lost to follow up: No colposcopy nor cytology	Y	National Health Service Cancer Screening Programmes Cervical Screening Call and Recall: Guide to Administrative Good Practice. (Canada 2014)	After 2 years after initial high grade Pap test	Cytobase (CytoBase has information on cervical cytology smears performed in community-based settings)	In person	Inadequate follow up: Cytology and no colp: ASCUS: 48.3% LSIL: 30.1% Colp and no repeat cytology: ASCUS: 15.7% LSIL: 26% Lost to follow-up: No cytology nor colposcopy ASCUS: 12.8% LSIL: 11.1%
Lindau (2006) [37]	Loss to follow up: No visit on chart review Inadequate follow up: Visit within a year of index Pap	N	(-)	Within a year of index Pap (Recommendation: 3 to 6 months depending on type of lesion)	Chart abstraction	Mixed (phone, mail and in person)	Lost to follow-up: 25% Followed within a year: 75%
Loopik (2020) [38]	Inadequate follow-up measured as a dichotomy (yes/no to having subsequent test (Yes/No)	Y	National Monitoring Cervical Cancer Screening (Netherlands/Dutch National Guidelines)	N	Nationwide network and registry of histo-and cytopathology in the Netherlands (PALGA)	N/A	No initial follow-up after abnormal test result: 8.1% No further follow-up after referral for colposcopy in clinician sampled group: 26% No further follow-up after referral to colposcopy in self sample group: 55.5%
Oladipo (2007) [39]	Failure to attend/cancelled to attending colposcopy appointment	N	(-)	N	Attendance, cancellation, and failure to attend were record by study	Phone	Appointment cancellation: Intervention: 7% Control: 18% Non-attendance: Intervention: 10% Control 24%
Percac-Lima (2013) [40]	Failure to attend appointment at Colposcopy clinic	Unclear	(-)	N	EMR chart review	Mixed (phone, visit and in person)	Control arm: 2004–2007 = 18.6% 2008–2011 = 20.6% (p= 0.45) Intervention: 2004–2007 = 19.8% 2008–2011 = 15.7% (p= 0.024)
Peterson (2003) [41]	Lack of a subsequent cervical cytology or pathology specimen within four months	Y	Definition operationalized by the study using various sources	4 months of the initial abnormal specimen for a high-grade lesion (HGSIL) or within seven months for a low-grade lesion (ASCUS, AGUS, or LGSIL).	Academic medical centre’s computerized pathology database	N/A	Inadequate follow-up: 38%
Salyer 2021 [42] ^b^ Salyer 2022 [47] ^b^	Self-reported: “Did you get the recommended follow-up after your Pap test”	N	(-)	N	Study Survey	Self-report	31% (18/58)
TOMBOLA Group (2009) [43]	Cytology surveillance non-attenders: if they did not attend for a cytology test or attended more than six months after it was due. In the immediate colposcopy arm, non-attenders failed to attend the two appointments offered	Y	National Health Service (NHS) Screening and follow-up Guidelines	6 months from recommended follow-up	Trial data-base and women medical records, together with hospital and pathology databases	In person	Non-attendance: Cytological surveillance arm 10.6% Immediate colposcopy: 6.8%
Tse (2016) [44]	Patients who did not attend cervical smear screening	Unclear	(-)	3 months from scheduled follow-up	Patients records and retrospective database review (Woo Women's Diagnostic and Treatment Centre (WDTC)	N/A	41.8% did not attend > = 1 follow-up screening. 20.7% did not return for follow-up
UshaKiran (2002) [45]	Failure attend colposcopy appointment	N	(-)	No	Clinic’s computer database	N/A	5.4% (37/685)
Valdini (2001) [46]	Unclear	Unclear	(-)	Unclear	Chart review	N/A	Lost to follow-up: 5.8% in control group (3/52)

^**a**^Outcome data extracted for patients > 20 years old only

^**b**^Study described in two publications

*NR* Not reported

The source of the definition for inadequate follow up varied. Local or national guidelines informed the definition in 58.3% of studies [21–24, 26, 29, 33–36, 38, 41, 43, 47]. Seven of the included studies (30%) [25, 28, 37, 39, 42, 45, 47] used their own definition. Studies conducted in the USA used a variety of sources to establish their definitions, such as local guidelines, [26] or guidelines from different academic associations such as the National Cancer Institute, [21] the American Society of Colposcopy and Cervical Pathology [24] and the American Cancer Society [33]. Others formed their definition from a range of different evidence sources.

Time to follow-up after a positive/abnormal test result was specified in 13 studies and ranged from 2.5 months [24] to 2 years [21, 36] with a median of 6 months (IQR: 9). Source of data to identify adequate follow-up was either through EMR or chart review or national or local databanks.

### Rates of inadequate follow-up.

Depending on the study design and country of origin, inadequate follow-up up ranged from 4% in a case control study from Canada, [25] to 75% in a cohort study from the USA, as shown in Table 2 [37].

Given the variety of the study types, the variability in the definitions of inadequate follow-up and the heterogeneity of results, we also chose to summarise prevalence according to study type in Table 3.

**Table 3:** Inadequate follow up by type of study^**a**^

Author	Study Type	Rates of inadequate follow up		Rates of no follow up	
		Intervention	Control	Intervention	Control
Brewer, 2021 [23]	RCT	(-)	(-)	Home self-sampling: 0 Clinic self-sampling: 8%	No inadequate follow-up
Breitkopf, 2014 [22]		Delays in days: 58 +-75	Delays in days 69 + -72 (p 0.75%)	22%	Active control: 25% Usual Care: 24% (p=0.93)
Engelstad, 2005 [26]		(-)	(-)	18.0%	20.0%
Gok, 2010 [29]		(-)	(-)	5.5% to 61.5%	(-)
Kristiansen, 2019 [35]		31.4%	35% (*p* < 0.005)	42.9%	47.2% (*p* < 0.005)
Oladipo, 2007 [39]		(-)	(-)	Appointment cancellation: 7% Non-attendance: 10.0%	Appointment cancellation: 18% Non-attendance: 24.0%
TOMBOLA Group 2009 [43]		(-)	(-)	6.8%	10.6%
Bernard 2005 [21]	Cohort	28.30%		27.70%	
Chase 2012 [24]		(-)		50%	
Gultekin 2018 [31]		(-)		(-)	
Kristiansen 2017 [34]				6 months (before) 64% (After) 62% (OR: 0.94 (0.90 to 0.97)) 3 months: (before) 62% (after) 60% (OR: 0.94 (0.90 to 0.97)) Within 3 months, (before) 16% (after) 16% (OR: 0.99 (0.95 to 1.02))	
Lindau 2006 [37]		75%		25%	
Peterson 2003 [41]		(-)		38%	
Tse 2016 [44]		41.80%		20.70%	
Felix 2009 [27]	Cross sectional	(-)		63.2%	
Hui 2014 [32]		N/A		N/A	
Kupets 2014 [36]		15.7%-48.3%		11.1–12.8%	
Salyer 2021- 2022^b^ [42, 47]		(-)		31% (p=0.144)	
Valdini 2001 [46]	Case series	(-)		5.7% in control group	
Dunn 2013 [25]	Non-randomised experimental study	13%		4% (*p* < 0.001)	
Fish 2013 [28]	Case Control	N/A		N/A	
UshaKiran 2002 [45]		(-)		5.4%	
Loopik 2020 [38]	Diagnostic accuracy	(-)		8.1- 55.5%	
Percac-Lima 2013 [40]	Non-randomised experimental study	(-)		Control arm: 2004–2007 = 18.6% 2008–2011 = 20.6% (p=0.45) Intervention: 2004–2007 = 19.8% 2008–2011 = 15.7% (p=0.024)	

^a^Outcome measures provided when available

^b^Study described in two publications

The lowest rates found were reported by Dunn et al. in a pre-post study measuring adherence of an on-site diagnostic colposcopy clinic [25]. The pre-intervention rates for inadequate follow-up were 13% which decreased to 4% post intervention (*P* < 0.001).

Prevalence for cohort studies ranged from 20.7% [44] to 75%. [37] Four studies [21, 31, 34, 44] were population-based and reported rates of inadequate follow-up from nationwide screening programmes, ranging from 28.3% [21] to 62% after a reminder message [34]. Chase, Lindau and Peterson [24, 37, 41] reported inadequate follow up for their clinic-based sample of women and their prevalence ranged from 38 to 75%. Prevalence for other types of studies is summarised in Table 3. We could not identify a specific trend throughout the years (increase or decrease of inadequate follow-up as technology improved).

### Factors associated with inadequate follow-up

Factors associated with inadequate follow-up were examined in 16 of the included studies [21, 22, 24–28, 34–37, 40–42, 44, 45, 47]. A total of 41 factors associated with inadequate follow-up of abnormal cervical screening were classified into 4 domains; patient, clinical, provider and system (Table 4). For each factor, studies were grouped according to statistical significance of the association with inadequate follow-up (positive, negative, or non-significant).

**Table 4:** Factors associated with inadequate follow-up

	**Factors**	**Positively associated (*p* > 0.05)**	**Non-significant**	**Negatively associated (*p* > 0.05)**
Patient	Younger Age	Dunn, 2013 [25]; Percac-Lima, 2013 [40]; Peterson, 2003 [41]; Tse, 2016 [44]; Usha Kiran, 2002 [45]Dunn, 2013 [25]; Percac-Lima, 2013 [40]; Peterson, 2003 [41]; Tse, 2016 [44]; Usha Kiran, 2002 [45]	Chase, 2012 [24]; Engelstad, 2005 [26]; Kupets, 2014 [36]; Salyer, 2021–22 [42, 47]	Benard, 2005 [21]; Felix, 2009 [27]; Kristiansen, 2017 [34];
	Married		Kristiansen, 2017 [34]; Percac-Lima, 2013 [40]; Tse, 2016 [44]; Salyer, 2021–22 [42, 47]Kristiansen, 2017 [34]; Percac-Lima, 2013 [40]; Tse, 2016 [44]; Salyer, 2021–22 [42, 47]	Chase, 2012 [24]
	Tertiary Education		Salyer, 2021–22 [42, 47]	Felix, 2009 [27]; Fish, 2013 [28]; Kristianson, 2017 [34]; Lindau, 2006 [37]
	High SES (household income)		Kupets, 2014 [36]	
	White Race (or country majority)		Engelstad, 2005 [26]; Felix, 2009 [27]; Peterson, 2003 [41]; Salyer, 2021–22 [42, 47]	Benard, 2005 [21]; Fish, 2013 [28]; Kristiansen, 2017 [34]
	English Language (or country majority)		Chase, 2012 [24]; Engelstad, 2005 [26]; Peterson, 2003 [41]	
	Privately insured		Dunn, 2013 [25]; Engelstad, 2005 [26]; Felix, 2009 [27]; Fish, 2013 [28]; Salyer, 2021–22 [42, 47]	Chase, 2012 [24]; Percac-Lima, 2013 [40]; Tse, 2016 [44]Chase, 2012 [24]; Percac-Lima, 2013 [40]; Tse, 2016 [44]
	Good/excellent self-perceived health status	Felix, 2009 [27]	Fish, 2013 [28]; Percac-Lima, 2013 [40]; Salyer, 2021–22 [42, 47]	
	High self-perceived competence/resources	Felix, 2009 [27]	Salyer, 2021–22 [42, 47]	
	Employment status, fulltime employed		Salyer, 2021–22 [42, 47]	
	Good/high knowledge of cervical/HPV		Lindau, 2006 [37]; Salyer, 2021–22 [42, 47]	Fish, 2013 [28]
	Rural/urban		Felix, 2009 [27]	
	Country of birth		Dunn 2013 [25]	
	Psychological symptoms	Breitkopf, 2014 (high depressive symptoms) [22]	Fish, 2013 [27]; Salyer 2021–22 (diagnosis of mental health issue) [42, 47]	Breitkopf, 2014 (high anxiety symptoms) [22]
	Trust in doctors		Felix, 2009 [27]	
	Current or past smoker	Tse, 2016 [44]	Salyer, 2021–22 [42, 47]	
	Self-reported as having barriers to screening		Salyer, 2021–22 [42, 47]	
	High QoL		Salyer, 2021–22 [42, 47]	
	Highly motivated to be healthy		Salyer, 2021–22 [42, 47]	
	High perception of severity of cervical cancer	Salyer, 2021–22 [42, 47]		
	High perception of susceptibility to cervical cancer	Salyer, 2021–22 [42, 47]		
	Time spent in jail/prison		Salyer, 2021–22 [42, 47]	
	History of abuse/violence (partner, childhood or neighbourhood)		Salyer, 2021–22 [42, 47]	
	Current or history of substance use		Salyer, 2021–22 [42, 47]	
Clinical	Obstetric status		Engelstad, 2005 [26]	
	Obstetric history, parity/gravidity > = 1		Tse, 2016 [44]; Usha Kiran, 2002 [44, 45]; Usha Kiran, 2002 [45]	Dunn, 2013 [25]
	Type of visit (screening)		Engelstad, 2005 [26]	Dunn, 2013 [25]
	Management plan/Intervention		Engelstad, 2005 [26]	Dunn, 2013 [25]
	History of regular screening			Percac-Lima, 2013 [40]
	Sexually-Transmitted Illness (STI) diagnosis		Salyer, 2021–22 [42, 47]	
	Past cancer diagnosis (non-cervical)		Salyer, 2021–22 [42, 47]	
	Received HPV vaccine	Salyer, 2021–22 [42, 47]		
Physician	Hormonal birth control use (compared to non-hormonal methods i.e., condoms)			Salyer, 2021–22 [42, 47]
	Past abnormal smear or HPV positive		Salyer, 2021–22 [42, 47]	
	Pathology (higher severity)		Dunn, 2013 [25]; Engelstad, 2005 [26]; Peterson, 2003 [41]; Usha Kiran 2002 [45]	Percac-Lima, 2013 [40]
	Profession/specialty in gynaecology		Peterson, 2003 [41]	Lindau, 2006 [37]
	Physician perception of patient literacy (low)	Lindau, 2006 [37]		
	Perception of patient likelihood to f/up (unlikely)		Lindau, 2006 [37]	
	Referral type—internal referral			Chase, 2012 [24]
System	Presence of regular primary care provider			Salyer, 2021–22 [42, 47]
	Direct notification of cervical cytology results			Kristiansen, 2019 [35]

^**a**^*Salyer results described in two publications* [42, 47]

^*b*^*Where both logistic regression and multi-level logistic regression were conducted, results from multi-level regression are presented*

### Patient factors

Most of the factors explored were patient factors, with 15/16 studies exploring at least one factor in this domain. The most explored factors were age (*n* = 12), [21, 24–27, 34, 36, 40–42, 44, 45, 47] insurance status (*n* = 8) [24–28, 40, 42, 44, 47] and race (*n* = 7) [21, 26–28, 34, 41, 42, 47]. The association between age and inadequate follow-up was inconsistent, with mixed findings across the studies. Insurance status and race were slightly more consistent, with most studies reporting negative associations with (or decreased rates of) inadequate follow-up for having private insurance and being white (or country majority). A detailed description of these associations can be found in Table 4.

**Fig. 3:**
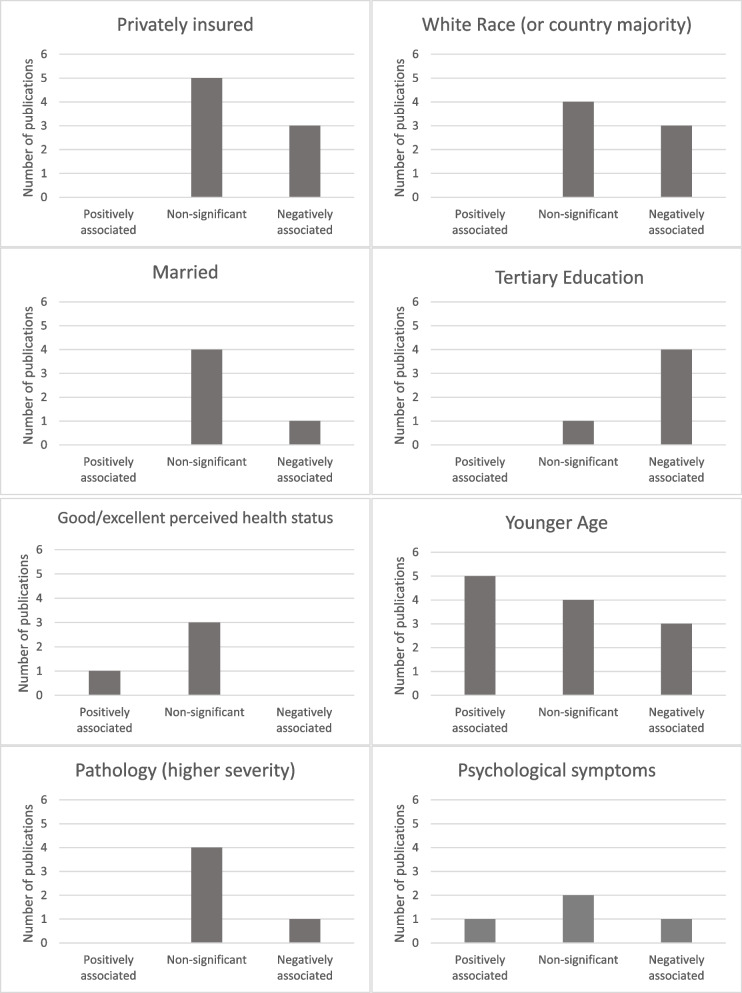
Most commonly assessed factors associated with inadequate follow-up

Psychological symptoms were explored in only three studies [22, 28, 42, 47]. Breitkopf et al*.* reported that high depressive symptoms were positively associated with inadequate follow-up, while high anxiety symptoms were associated with lower rates of inadequate follow-up [22]. Having a tertiary education was mostly associated with decreased rates of inadequate follow-up [22, 27, 28, 34, 37]. The associations of other frequent individual factors are summarised in Fig. 3.

### Clinical and provider factors

Clinical and provider factors were infrequently assessed. Factors such as type of visit or having a management plan showed inconsistent evidence of association [25, 26]. Dunn et al. found that women were significantly less adherent if they had been screened at an abortion clinic than if they had been referred directly after a Pap screening-specific appointment [25] while Engelstad et al*.* found the intervention to be equally successful if the reason for their initial Pap had been as a routine or a diagnostic appointment [26].

Severity of the lesion was measured in five different studies [25, 26, 40, 41, 45] and was not related to inadequate follow-up in all but one study [40].

### System factors

These were measured in three, more recent studies [24, 35, 42, 47]. Internal referral to colposcopy (from the same clinic), [24] presence of a regular health care provider, [42, 47] and direct notification to women of cervical abnormalities results, [35] were all associated with lower rates of inadequate follow-up [24, 35, 42, 47].

### Qualitative studies

We identified and analysed two qualitative studies [30, 33]. In exploring barriers and facilitators to follow-up, issues relating to communication were identified as a common theme in both studies. Goldsmith et al*.* explored women’s information needs for abnormal results and found the need for more didactic forms of information (diagrams) and the need to receive their results directly, with a knowledgeable intermediary other than the general practitioner (GP), outside primary care. Hunt et al*.* identified several barriers to follow-up including poor communication from the health care provider regarding their need to follow-up, mixed messages, getting follow-up care elsewhere, clinical errors, and inordinate follow-up requirements [33].

### Intervention to increase adherence to follow-up after receiving an abnormal cervical cancer screening test result

Even though the aim of the review was not to identify effective interventions for adherence, but rather rates of inadequate follow up, we identified 7 RCTs testing interventions to decrease inadequate follow up. All RCTs but one [22] (6/7) were effective. Two studies reported on the use of self-sampling to increase adherence to screening, both with positive findings. Brewer et al., reported that sending women a self-sample kit for HPV testing at home resulted in statistically significantly (*p* ≤ 0.001) higher participation than an invitation to have a usual-care cytology sample in the clinic among Maori women (14.6% vs. 2.0%) [23] and Gok et al. described a 27.5% of compliance vs a 16.6% in the intervention vs control groups respectively with a difference of compliance of 10.9% (*P* < 0.001) after adjustment for those who were not eligible [29].

Other effective interventions described were: A counselling and outreach intervention for improving rates of follow-up of abnormal Pap smears [26]; a pre-clinic telephone contact intervention for prospective patients in a colposcopy clinic [39]; cytological surveillance in primary care compared with immediate referral for colposcopy examination in women with low grade abnormal results on cervical cytology tests [43]; and direct notification to women of cervical cytology results on follow-up rates [35].

## Discussion

In this systematic review, we describe the prevalence of inadequate follow-up after receiving abnormal cervical screening results in the primary and ambulatory setting, and the factors affecting follow-up. We also summarise evidence found about interventions that have been shown to improve follow-up. Given the multiplicity of health systems around the world and the various definitions of what can be considered primary care, we opted for an inclusive approach and considered studies that were based in primary care as well as non-hospital based ambulatory care.

### Definitions of inadequate follow-up

Definitions for inadequate follow-up varied. Most studies defined inadequate follow-up as either a) non-attendance to a follow-up appointment and/or b) the absence of further testing at various time points after abnormal test result. Most studies outside the USA, used national screening guidelines to construct their definitions of inadequate follow-up. All of these countries have national cervical screening programs [4]. While the USA has a national screening program in place, it is executed by many different providers, from family medicine clinics to women’s health clinics to specialist colposcopy clinics. This multiplicity of providers may be one of the reasons why researchers feel compelled to use a variety of sources to define inadequate guidelines suitable to their particular settings.

### Prevalence of inadequate follow-up

Prevalence of inadequate follow-up varied substantially across the studies. In the studies conducted in the USA, the heterogeneity of definitions, different levels of risk of bias, along with the fact that follow-up in most studies were performed in a range of primary care/ambulatory settings, in very different populations, may account for the wide range of prevalence of inadequate follow-up encountered in the different studies.

Even though this wide range in the prevalence of inadequate follow-up of abnormal cervical cancer screening tests results is undoubtedly multifactorial, we hypothesise that differences in health systems and cancer screening programs may play an important role. According to the WHO, all locations mentioned in this review had a cancer screening program in place in 2021 [4]. Nevertheless, there are system differences that could explain the variation in rates. The USA, with the highest rates of inadequate follow-up, for example, does not have data available on whether programme/guidelines exist to strengthen early detection of first symptoms at primary health care level or whether a clearly defined referral system exists from primary care to secondary and tertiary care. All other locations report positively in these two aspects of their screening programs [4]. Likewise, all locations had programmes in place at the time of these studies [48–52]. However, for two of them, (Türkiye and Hong Kong) screening programs had newly begun. Türkiye started a cytology-based screening program in 2004, switching to HPV in 2014. [31] Hong Kong started theirs in 2004 [53]. The two studies conducted in the Netherlands reported inadequate follow-up for a colposcopy in 2020 was lower than in 2010. In 2017, the Netherlands replaced cytology testing for HPV. After their first HPV positive test, women are invited to perform a cytology test with their local GP [38]. This suggests that a comprehensive national screening program based in primary care and HPV testing is an effective way to increase adherence to follow-up after a positive result. Currently, of the countries in this study, only the Netherlands, United Kingdom and Türkiye have a program based on HPV screening. A systematic review on the impact of health systems’ influence on the speed of cancer diagnosis found that even though a causal correlation between healthcare system characteristics and cancer outcomes could not be found, factors such as centralisation of services, free movement of patients between primary care providers, access to secondary care, and the existence of patient list systems could impact cancer diagnosis delays and account for worse cancer outcomes in the countries studied [54]. Access to secondary care could be one of the reasons for the studies with low inadequate follow-up in this review. These were conducted in a women’s clinic in Canada. [25] and a colposcopy clinic in the UK [45]. They tested interventions to increase adherence by providing expedited access to colposcopy follow-up.

### Factors associated with inadequate follow-up

We explored factors associated with inadequate follow-up of a positive cervical test result in primary care. Most factors had no statistically significant association with inadequate follow-up and findings were inconsistent across studies. For example, younger age was positively associated with inadequate follow-up in five studies, negatively associated in three studies, and found to be non-significant in four studies.

Race, low socioeconomic status (SES), and lack of health insurance have long been associated with lower cancer screening rates and poorer cancer outcomes in women. In the USA, Latina women have the highest incidence of cervical cancer whereas African Americans have the higher mortality rates followed by Latinas. [55] In 2019, the American Cancer Society reported higher cervical cancer screening rates for white women (77%) than for African Americans (75%), Hispanics (67%), Asian (66%), and American-Indian/Alaska-natives (70%) and for women with private insurance (80%) vs uninsured (54%).Overall screening rates decreased significantly from 2005 to 2019 even before Covid in all populations with higher numbers in minority populations (From 14 to 23% overall). Even though lack of access to health care services and insurance status continue to be reasons for underscreening, from 2005 to 2019, lack of access decreased significantly as the primary reason (from 21.8% to 9.7%). Instead, lack of knowledge (from 45.2% to 54.8%) and not receiving recommendations from health care professionals (from 5.9% to 12.0%) increased significantly, making the former the main cause for underscreening in the USA [56]. Sadly, this has not improved much in the last 30 years, when for example, a systematic review of studies published between 1985 and 1999 reported patient factors such as lack of social support, lack of understanding, and fear as main reasons for underscreening [13]. This is also the case in low- and middle-income countries where cancer prevention and outcomes are worse than in high-income countries due to multiple factors including lack of access to health services, low income, and low education among others [57]. In our review, race, particularly being white, was negatively associated with inadequate follow-up in three of seven studies that reported this variable; low SES and private insurance were only associated in a few studies. This may be due to some studies not using population-based data, instead focusing on specific minority populations (Latina, Maori etc.) therefore, it may have altered the possible association between race, SES and inadequate follow-up.

Psychological factors were seldom explored, and results were contradictory. Depressive symptoms were described as a risk factor for inadequate follow-up while anxiety symptoms were said to be associated with lower rates of inadequate follow-up [22]. This suggests that psychological symptoms or diagnosis of mental health issues may require further granularity when being explored as a factor for follow-up. Fish et al. [28] and Salyer et al. [42, 47] did not find a statistically significant difference, this could be due to a small sample size or lack of statistical power. The qualitative studies in our search described patients feeling “panicked” and “shocked” when receiving abnormal test results however, they do not describe how those reactions relate to follow-up. Rather, the studies discuss system factors and patient-provider communication as reasons for inadequate follow-up [30, 33]. Although communication was a key theme in both qualitative studies, communication is not mentioned as a factor for inadequate follow-up in any of the quantitative studies; personal factors were the point of focus instead. Physician factors and system factors were least likely to be explored, with only three studies including system-level factors and two studies including physician-level factors [24, 35, 37, 41, 42, 47].

### Effective interventions to increase adherence of follow-up of abnormal cervical cancer screening

Although the primary aim of this study was not to identify effective interventions to increase follow-up of abnormal test results but rather describe factors that influence inadequate follow-up of abnormal cervical cancer screening in primary care, our search identified seven RCTs investigating different interventions to increase follow-up [22, 23, 26, 29, 35, 39, 43].

Positive results were seen by reducing barriers to access and by asking patients to self-sample or providing test results directly to the patients among others [23, 35]. These results are consistent with evidence that system-based interventions and interventions addressing patient and provider behaviours have proven effective in increasing adequate follow-up of abnormal cervical screening results in primary care [14].

One RCT evaluated cytological surveillance at 6 months performed in primary care compared with immediate referral for colposcopy at a hospital outpatient clinic for management of women with low grade cervical abnormalities [43]. Rates of inadequate follow-up were higher in the cytological surveillance arm compared to the ones referred for immediate colposcopy. A systematic review found evidence that women with abnormal tests preferred active follow-up, predominantly as immediate colposcopy, to observation and/or to repeated Pap smears. In this case, setting might be less important than the anxiety produced by the abnormal result and so women might be more willing to attend an immediate colposcopy appointment rather than observation in primary care [58].

### Limitations

This review is limited by the lack of data from low- and middle-income countries. All of the studies identified in this search were conducted in high- or upper-middle income countries [59]. Inadequate follow-up is higher in middle- income/low-income countries and account for about 90% of new cases and deaths worldwide in 2020 [59, 60]. The existence of national screening programs facilitates the reporting of such statistics, yet we are unable to calculate the same prevalence for middle/low-income countries without organised cervical cancer screening programs. We had to exclude 14 studies for which there were no English full texts available. This may bias the information to represent only studies that can report their results in English (usually, again, high income countries).

There is also the risk of publication bias, as those studies with positive results might be more likely to be accepted for publication. During the initial screening for this review, we found most studies report on adherence to follow-up instead of inadequate follow-up, this limited the number and the quality of the information provided. We were unable to perform a meta-analysis given the heterogeneity of follow-up definitions and quality of included studies.

### Deviation from protocol

One of our original aims was to investigate: “what are the adverse effects of inadequate follow-up?”. Once we started to investigate the literature more closely and developing the extraction tables, we decided that describing adverse effects of inadequate follow-up was outside of the scope of this review. There is strong evidence of the adverse effect inadequate follow-up poses to cancer outcomes [7, 11, 12]. Future reviews could investigate the psychosocial effects and the burden on quality of life caused by decreased compliance to cervical cancer screening.

## Conclusion

Cervical cancer is the fourth leading cause of cancer death for women around the world. Primary care is defined as the first point of contact for health care and yet, there is incomplete and inconsistent evidence on the prevalence and factors that affect inadequate follow-up in primary care. We found that the prevalence and definitions of inadequate follow-up in primary care vary, and information regarding factors associated with it is contradictory. All information available comes from high- or upper-middle income countries with established screening programs with different levels of success. Further research is needed on factors that affect inadequate follow-up of positive cervical screening results in low- and middle-income countries without regular screening programs in place. Evidence of effective interventions to improve follow-up should be implemented more widely to improve outcomes for cervical cancer.

## Supplementary Information

**Additional file 1.** 

**Additional file 2.** 

## Data Availability

All data generated or analysed during this study are included in this published article [and its supplementary information files].
