# Supplementary material for: Failure to follow up abnormal test results associated with cervical cancer in primary and ambulatory care: a systematic review
Source: BMC Cancer. 2023 Jul 12;23:653. doi: 10.1186/s12885-023-11082-z (PMC10337158; doi:10.1186/s12885-023-11082-z)
Supplement: Supplementary file 1 — Additional file 1. [file 12885_2023_11082_MOESM1_ESM.docx]

**Appendix I. Search strategy**

**MEDLINE, Embase and Cochrane libraries (Ovid)**

General practice/ OR Family practice/ OR Physicians, Family/ OR Physicians, Primary Care/ OR General practitioners/ OR Primary Health Care/ OR Ambulatory care/

(General practi* OR primary care OR primary healthcare OR primary health care OR family physician* OR ambulatory care* OR outpatient care*).ti,ab.

*AND*

Medical errors/ OR Delayed diagnosis/ OR Diagnostic Errors/ OR Incidental findings/ OR Clinical decision-making/

(Error* OR delay* OR miss OR missed OR misdiagnos* OR time*).ti,ab.

(Follow up OR flag up OR red flag OR flag* abnormal).ti,ab.

(Patient safety OR quality improvement).mp.

*AND*

Papanicolaou Test/ or vaginal smear/ or cervical smear/ or (pap smear* or pap* test*).ti,ab.

Human papillomavirus/ or Human papillomavirus dna test*/ or papillomavirus.mp. or (hpv and test*).ti,ab.

Colposcopy/ OR (Colposcop*).ti,ab

Uterine Cervical Neoplasms/ or Uterine Cervical Dysplasia/ or Cervical Intraepithelial Neoplasia/ or cervical cancer.mp

** = Truncation (finding all terms that begin with the given string of text)*

*.ti.ab = searches for term in title & abstract*

*.mp = multiple field search (title, abstract, original title, name of substance word, subject heading word, keyword heading word, protocol supplementary concept word, rare disease supplementary concept word, unique identifier, synonyms)*

*exp = exploded thesaurus (picks up all articles that contain the term as a keyword)*
