# Supplementary material for: Failure to follow up abnormal test results associated with cervical cancer in primary and ambulatory care: a systematic review
Source: BMC Cancer. 2023 Jul 12;23:653. doi: 10.1186/s12885-023-11082-z (PMC10337158; doi:10.1186/s12885-023-11082-z)
Supplement: Supplementary file 2 — Additional file 2. [file 12885_2023_11082_MOESM2_ESM.docx]

Appendix 2. Joanna Briggs Clinical Assessment Tools.

Joanna Briggs Critical Appraisal Checklist for Randomized Control Trials [yes, no, unclear, not applicable]

| Author | 1 | 2 | 3 | 4 | 5 | 6 | 7 | 8 | 9 | 10 | 11 | 12 | 13 | Quality |
| --- | --- | --- | --- | --- | --- | --- | --- | --- | --- | --- | --- | --- | --- | --- |
| Breitkopf | Yes | Yes | Yes | Yes | N/A | Yes | Yes | Yes | Yes | Yes | Yes | Yes | Yes | 100.0 |
| Engelstad | Yes | Unclear | Yes | N/A | N/A | Unclear | Yes | Yes | Yes | Yes | Yes | Yes | Unclear | 72.7 |
| Gok | Yes | Yes | Unclear | N/A | N/A | Unclear | Yes | Yes | Yes | Yes | Yes | Yes | Unclear | 72.7 |
| Kristiansen | Yes | Yes | Yes | N/A | Unclear | Unclear | Unclear | Yes | Yes | Yes | Yes | Yes | Yes | 75.0 |
| Oladipo | Unclear | Unclear | Yes | N/A | Unclear | Unclear | Yes | Yes | Unclear | Yes | Unclear | No | Unclear | 33.3 |
| TOMBOLA group | Yes | Yes | Yes | N/A | N/A | Yes | Yes | Yes | Yes | Yes | Yes | Yes | Yes | 100.0 |
| Brewer | Yes | Yes | Yes | N/A | N/A | Unclear | Yes | Yes | Yes | Yes | Yes | Yes | Yes | 90.9 |

1. Was true randomization used for assignment of participants to treatment groups? 2. Was allocation to treatment groups concealed? 3. Were treatment groups similar at the baseline? 4. Were participants blind to treatment assignment? 5. Were those delivering treatment blind to treatment assignment? 6. Were outcomes assessors blind to treatment assignment? 7. Were treatment groups treated identically other than the intervention of interest? 8. Was follow up complete and if not, were differences between groups in terms of their follow up adequately described and analyzed? 9. Were participants analyzed in the groups to which they were randomized? 10. Were outcomes measured in the same way for treatment groups? 11. Were outcomes measured in a reliable way? 12. Was appropriate statistical analysis used? 13. Was the trial design appropriate, and any deviations from the standard RCT design (individual randomization, parallel groups) accounted for in the conduct and analysis of the trial?

Joanna Briggs Critical Appraisal Checklist for Quasi Experimental Trials [yes, no, unclear, not applicable]

| Author | 1 | 2 | 3 | 4 | 5 | 6 | 7 | 8 | 9 | Quality |
| --- | --- | --- | --- | --- | --- | --- | --- | --- | --- | --- |
| Dunn | Yes | No | Unclear | Yes | N/A | Yes | Yes | Unclear | Yes | 62.5 |
| Percac-Lima | Yes | Yes | Unclear | Yes | N/A | Yes | Yes | Yes | Yes | 87.5 |

"1. Is it clear in the study what is the ‘cause’ and what is the ‘effect’(i.e. there is no confusion about which variable comes first)? 2. Were the participants included in any comparisons similar? 3. Were the participants included in any comparisons receiving similar treatment/care, other than the exposure or intervention of interest? 4. Was there a control group? 5. Were there multiple measurements of the outcome both pre and post the intervention/exposure? 6. Was follow up complete and if not, were differences between groups in terms of their follow up adequately described and analyzed? 7. Were the outcomes of participants included in any comparisons measured in the same way? 8. Were outcomes measured in a reliable way? 9. Was appropriate statistical analysis used?

Joanna Briggs Critical Appraisal Checklist for Cohort Studies [yes, no, unclear, not applicable]

| Author | 1 | 2 | 3 | 4 | 5 | 6 | 7 | 8 | 9 | 10 | 11 | Quality |
| --- | --- | --- | --- | --- | --- | --- | --- | --- | --- | --- | --- | --- |
| Benard | Yes | Yes | Yes | No | No | Yes | Yes | Unclear | No | Yes | N/A | 60.0 |
| Chase | Yes | Yes | Yes | Yes | Yes | Yes | Yes | Yes | Yes | Unclear | Unclear | 81.8 |
| Gultekin | N/A | N/A | Unclear | N/A | N/A | Yes | Yes | Unclear | Unclear | No | No | 25.0 |
| Kristiansen | Yes | Yes | Yes | Yes | Yes | Yes | Yes | Yes | Yes | Yes | Yes | 100.0 |
| Lindau | Yes | Yes | Yes | Yes | Yes | Yes | Unclear | Unclear | Yes | Yes | Unclear | 72.7 |
| Peterson | Yes | Yes | Yes | Yes | Yes | Yes | Yes | Yes | Yes | Yes | Yes | 100.0 |
| Tse | Yes | Yes | Yes | Yes | Yes | Yes | Yes | Yes | Yes | Yes | Yes | 100.0 |

1. Were the two groups similar and recruited from the same population? 2. Were the exposures measured similarly to assign people to both exposed and unexposed groups? 3. Was the exposure measured in a valid and reliable way? 4. Were confounding factors identified? 5. Were strategies to deal with confounding factors stated? 6. Were the groups/participants free of the outcome at the start of the study (or at the moment of exposure)? 7. Were the outcomes measured in a valid and reliable way?" 8. Was the follow up time reported and sufficient to be long enough for outcomes to occur? 9. Was appropriate statistical analysis used? 10. Was follow up complete, and if not, were the reasons to loss to follow up described and explored? 11. Were strategies to address incomplete follow up utilized?

Joanna Briggs Critical Appraisal Checklist for Cross sectional Studies [yes, no, unclear, not applicable]

| Author | 1 | 2 | 3 | 4 | 5 | 6 | 7 | 8 | Quality |
| --- | --- | --- | --- | --- | --- | --- | --- | --- | --- |
| Felix | Yes | Yes | Unclear | No | Yes | Yes | Unclear | Unclear | 50.0 |
| Hui | Yes | Yes | Yes | Yes | Unclear | Unclear | Yes | Unclear | 62.5 |
| Kupets | Yes | No | Yes | Yes | Unclear | Unclear | Yes | Unclear | 50.0 |
| Salyer | Yes | Yes | Yes | No | Yes | Yes | Unclear | Yes | 75.0 |
| Salyer | Yes | Yes | Yes | No | Yes | Yes | Unclear | Yes | 75.0 |

1. Were the criteria for inclusion in the sample clearly defined? 2. Were the study subjects and the setting described in detail? 3. Was the exposure measured in a valid and reliable way? 4. Were objective, standard criteria used for measurement of the condition? 5. Were confounding factors identified? 6. Were strategies to deal with confounding factors stated? 7. Were the outcomes measured in a valid and reliable way? 8. Was appropriate statistical analysis used?

Joanna Briggs Critical Appraisal Checklist for Case Control studies[yes, no, unclear, not applicable]

| Author | 1 | 2 | 3 | 4 | 5 | 6 | 7 | 8 | 9 | 10 | Quality |
| --- | --- | --- | --- | --- | --- | --- | --- | --- | --- | --- | --- |
| Fish | No | N/A | Yes | Yes | Yes | Yes | Yes | Yes | Unclear | Yes | 77.8 |
| UshaKiran | Yes | Yes | Yes | Yes | Yes | Unclear | Unclear | Yes | Unclear | Yes | 77.8 |

1. Were the groups comparable other than the presence of disease in cases or the absence of disease in controls? 2. Were cases and controls matched appropriately? 3. Were the same criteria used for identification of cases and controls? 4. Was exposure measured in a standard, valid and reliable way? 5. Was exposure measured in the same way for cases and controls? 6. Were confounding factors identified? 7. Were strategies to deal with confounding factors stated? 8. Were outcomes assessed in a standard, valid and reliable way for cases and controls? 9. Was the exposure period of interest long enough to be meaningful? 10. Was appropriate statistical analysis used?

Joanna Briggs Critical Appraisal Checklist for Qualitative Studies [yes, no, unclear, not applicable]

| Author | 1 | 2 | 3 | 4 | 5 | 6 | 7 | 8 | 9 | 10 | Quality |
| --- | --- | --- | --- | --- | --- | --- | --- | --- | --- | --- | --- |
| Goldsmith | Yes | Yes | Yes | Yes | Yes | No | Unclear | Yes | Yes | Yes | 80.0 |
| Hunt | Unclear | Yes | Yes | Yes | Yes | Yes | Unclear | Yes | Yes | Yes | 80.0 |

1. Is there congruity between the stated philosophical perspective and the research methodology? 2. Is there congruity between the research methodology and the research question or objectives? 3. Is there congruity between the research methodology and the methods used to collect data? 4. Is there congruity between the research methodology and the representation and analysis of data? 5. Is there congruity between the research methodology and the interpretation of results? 6. Is there a statement locating the researcher culturally or theoretically? 7. Is the influence of the researcher on the research, and vice- versa, addressed? 8. Are participants, and their voices, adequately represented? 9. Is the research ethical according to current criteria or, for recent studies, and is there evidence of ethical approval by an appropriate body? 10. Do the conclusions drawn in the research report flow from the analysis, or interpretation, of the data?

Joanna Briggs Critical Appraisal Checklist for Case Series [yes, no, unclear, not applicable]

| Author | 1 | 2 | 3 | 4 | 5 | 6 | 7 | 8 | 9 | 10 | Quality |
| --- | --- | --- | --- | --- | --- | --- | --- | --- | --- | --- | --- |
| Valdini | Yes | Yes | Yes | Yes | Yes | No | Yes | Yes | Yes | No | 80.0 |

"1. Were there clear criteria for inclusion in the case series? 2. Was the condition measured in a standard, reliable way for all participants included in the case series? 3. Were valid methods used for identification of the condition for all participants included in the case series? 4. Did the case series have consecutive inclusion of participants? 5. Did the case series have complete inclusion of participants? 6. Was there clear reporting of the demographics of the participants in the study? 7. Was there clear reporting of clinical information of the participants? 8. Were the outcomes or follow up results of cases clearly reported? 9. Was there clear reporting of the presenting site(s)/clinic(s) demographic information? 10. Was statistical analysis appropriate?

Joanna Briggs Critical Appraisal Checklist for Diagnostic Accuracy [yes, no, unclear, not applicable]

| Author | 1 | 2 | 3 | 4 | 5 | 6 | 7 | 8 | 9 | 10 | Quality |
| --- | --- | --- | --- | --- | --- | --- | --- | --- | --- | --- | --- |
| Loopik | No | Yes | Yes | Yes | N/A | Yes | Yes | Yes | Yes | Yes | 88.9 |

"1. Was a consecutive or random sample of patients enrolled? 2. Was a case control design avoided? 3. Did the study avoid inappropriate exclusions? 4. Were the index test results interpreted without knowledge of the results of the reference standard? 5. If a threshold was used, was it pre-specified? 6. Is the reference standard likely to correctly classify the target condition? 7. Were the reference standard results interpreted without knowledge of the results of the index test? 8. Was there an appropriate interval between index test and reference standard? 9. Did all patients receive the same reference standard? 10. Were all patients included in the analysis?
